# Peer Review of “Use of Spinal Anesthesia in Pediatric Laparoscopic Appendectomies: Case Series”

**DOI:** 10.2196/29605

**Published:** 2021-04-28

**Keywords:** pediatrics, appendectomy, spinal anesthesia, general anesthesia, laparoscopy, vomiting, keyhole, surgery, anesthesia, appendix

*This is a peer-review report submitted for the paper “Use of Spinal Anesthesia in Pediatric Laparoscopic Appendectomies: Case Series.”*

## Round 1 Review

### General Comments

The authors of this paper [1] should be commended for their hard work in advocating for the interesting and potentially beneficial yet underused practice of neuraxial spinal anesthesia in the pediatric population for laparoscopic surgery. While this is certainly a topic worthy of additional research and publication, this report in its current form presents several significant challenges that will need to be addressed prior to acceptance for publication. While it is certainly understandable for the authors to attempt to demonstrate the potential benefit of this technique compared with the standard-of-care general anesthetic, I am concerned that the data as presented (or lack thereof) render this less appropriate as a case-control study and more appropriately a case-series report (describing the experience and outcomes of patients undergoing the spinal technique, not comparing them against patients undergoing a general anesthetic). In the absence of randomized control, and without describing a protocol for how the anesthetic technique was decided, the possibility of confounding factors becomes unacceptably large when attempting to draw conclusions from a sample size of this magnitude.

Additional information that can be provided to strengthen case-control studies, which this manuscript could benefit from (see Moola et al [2]) include:

1. Were the groups comparable apart from the choice of anesthetic (and age)—were underlying medical conditions, weight, family history of postoperative nausea and vomiting, developmental history similar?
2. Was the presence of postoperative nausea or vomiting a binary measure?

### Specific Comments

#### Major Comments

The focus on the incidence of postoperative nausea or vomiting between the spinal and general anesthetic groups is particularly problematic given what is described regarding the protocol (and the authors’ own admission in the *Discussion* section, which states “confounding factors from different adjuncts delivered intraoperatively make these results somewhat more difficult to interpret—in fact, the entire subject of postoperative nausea and vomiting can be quite complex”). Even without a significant description of the protocols (both experimental and anesthetic) provided, questions can be raised about the construction of the study. Patients undergoing spinal anesthesia received sedation with diazepam and ketamine (both drugs with a long duration of effects and antiemetic properties), while patients undergoing general anesthesia had nitrous oxide (and possibly a volatile agent?) throughout the duration of the case—a fact that alone is likely to put that patient population at significant risk for postoperative nausea or vomiting. The fact that a number of additional analgesics, antiemetics (which antiemetics were administered to all patients—ondansetron or another agent?), and adjuncts may be given by a number of different providers raises the potential for significant confounding of these measures.

#### Minor Comments

1. There are minor grammatical and sentence construction choices that would benefit from additional copyediting.
2. What currency is used in describing the cost of the procedure?
